# Towards a tunable graphene-based Landau level laser in the terahertz regime

**DOI:** 10.1038/srep12646

**Published:** 2015-07-29

**Authors:** Florian Wendler, Ermin Malic

**Affiliations:** 1 Chalmers University of Technology, Department of Applied Physics, SE-412 96 Göteborg, Sweden

## Abstract

Terahertz (THz) technology has attracted enormous interest with conceivable applications ranging from basic science to advanced technology. One of the main challenges remains the realization of a well controlled and easily tunable THz source. Here, we predict the occurrence of a long-lived population inversion in Landau-quantized graphene (i.e. graphene in an external magnetic field) suggesting the design of tunable THz Landau level lasers. The unconventional non-equidistant quantization in graphene offers optimal conditions to overcome the counteracting Coulomb- and phonon-assisted scattering channels. In addition to the tunability of the laser frequency, we show that also the polarization of the emitted light can be controlled. Based on our microscopic insights into the underlying many-particle mechanisms, we propose two different experimentally realizable schemes to design tunable graphene-based THz Landau level lasers.

In 1986 H. Aoki proposed the first Landau level laser for two-dimensional electron systems^1^ exploiting the discreteness of the Landau levels (LLs) to tune the laser frequency through the magnetic field. The key challenge for the realization of such a LL laser is to obtain and to sustain a long-lived population inversion (PI) between LLs. This is difficult to achieve in conventional semiconductors where strong Coulomb scattering between equidistant LLs acts in favor of an equilibrium Fermi-Dirac distribution. Similarly, phonon-induced scattering can counteract the population inversion, if the phonon energy is in resonance with the inter-Landau level transitions involved in the lasing process.

Graphene as a two-dimensional zero-gap semiconductor with remarkable properties^2^ offers optimal conditions for LL lasing. Its linear electronic dispersion leads to an unconventional non-equidistant LL spacing including the appearance of a zero Landau level in an external magnetic field^3^,^4^. The observation of a number of interesting effects such as the fractional quantum Hall effect^5^,^6^, a giant Faraday rotation^7^, the quantum ratchet effect^8^, the Hofstadter butterfly^9^, and the demonstration of a tunable THz detector^10^ has already attracted enormous interest to Landau-quantized graphene^11^. The non-equidistant LLs and the specific optical selection rules allowing transitions between LLs with *n* → *n* ± 1 have been predicted to make graphene an optimal material for the realization an efficient two-dimensional LL laser^12^. A transient population inversion in graphene without a magnetic field has already been theoretically predicted^13^,^14^ and experimentally demonstrated^15^,^16^,^17^,^18^. It emerges as a result of a relaxation bottleneck close to the Dirac point and decays mainly due to Coulomb-induced recombination processes^14^.

In this article, we predict the occurrence of a long-lived population inversion in Landau-quantized graphene. We present two different experimentally feasible mechanisms to achieve the population inversion induced by optical pumping, cf. Fig. 1. The first mechanism (A) is based on the specific optical selection rules in Landau-quantized graphene yielding the possibility to selectively pump a single LL transition constituting an effectively three-level laser system, cf. Fig. 1A. Using a linearly polarized optical excitation field with an energy matching the inter-Landau level transition LL_−3_ → LL_+2_ (and LL_−2_ → LL_+3_), a population inversion between LL_+1_ and LL_+2_ (*σ*^+^-PI) (and between and between LL_−2_ and LL_−1_ (*σ*^−^-PI)) is generated. The second mechanism (B) exploits the scattering among electrons to achieve PI and thereby adds an additional level to the system, which can be beneficial for the efficiency of the laser, cf. Fig. 1B. Here, a linearly polarized pump pulse resonantly exciting the transitions LL_−7_ → LL_+8_ and LL_−8_ → LL_+7_ is used to induce Auger scattering that generates a population inversion between the same levels as in scheme A by populating LL_+2_ and depopulating LL_−2_. Interestingly, scheme B provides a Coulomb-induced mechanism to create PI, which is quite remarkable, because Coulomb-induced Auger scattering was shown to rather reduce PI in graphene^14^ and has been believed to be the main obstacle for the realization of a graphene-based two-dimensional LL laser^19^,^20^. Since we preserve the electron-hole symmetry, PI is obtained in the conduction band and in the valence band at the same time. It occurs between the LLs with the indices *n* = 1 and *n* = 2. However, we want to stress that also other schemes are possible to obtain PI for different LL transitions. Note that the PI transitions in the conduction and in the valence band are optically coupled by inversely circularly polarized photons, i.e. photons created in a stimulated emission process inducing the electronic transition LL_+2_ → LL_+1_ (LL_−1_ → LL_−2_) are *σ*^+^- polarized (*σ*^−^- polarized). Hence, we label the corresponding population inversion as *σ*^+^- PI and *σ*^−^- PI, respectively.

## Results and Discussion

While the carrier dynamics without a magnetic field has been already thoroughly studied in experiment^17^,^21^,^22^,^23^,^24^ and theory^14^,^24^,^25^,^26^,^27^,^28^,^29^, its investigation in Landau-quantized graphene has just started to pick up pace very recently^19^,^30^,^31^,^32^. We have developed a theory based on the density matrix approach^29^ providing access to time and energy-dependent relaxation dynamics in Landau-quantized graphene and revealing microscopic insights into the underlying many-particle scattering pathways^30^,^31^. The temporal evolution of LL carrier occupations 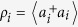 and microscopic polarizations 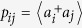 (with the fermionic creation and annihilation operators 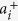 and *a*_*i*_) determining the strength of optical LL transitions is obtained using the graphene Bloch equations in the presence of a magnetic field


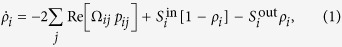



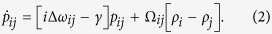


The set of coupled differential equations has been obtained by exploiting a correlation expansion within the second-order Born-Markov approximation^29^. Here, we explicitly take into account the occupations and polarizations of the energetically lowest LLs up to *n* = 10, including the optical excitation as well as all energy-conserving carrier-carrier and carrier-phonon scattering processes. The Rabi frequency 
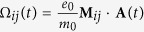
 appearing in Eqs (1) and (2) depends on the electron’s charge *e*_0_, its free mass *m*_0_, the optical matrix element **M**_*ij*_, and on the vector potential **A**(*t*). The scattering rates 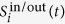 incorporate all energy-conserving electron-electron and electron-phonon scattering processes including time-dependent Pauli blocking terms. The Coulomb interaction is dynamically screened taking into account the momentum dependence of the dielectric function in the random phase approximation^11^,^31^. Phonon-induced scattering via the dominant optical phonon modes ΓTO, ΓLO, and KTO is included, where a coupling to a bath is considered. The microscopic polarization decays due to a dephasing *γ*(*t*) caused by many-particle scattering as well as impurity-induced LL broadening. The energy difference Δ*ω*_*ij*_ = (*ε*_*i*_ − *ε*_*j*_)/*ħ* between LL_*i*_ and LL_*j*_ describes the oscillation of the corresponding polarization. Excitons are not considered, since no signatures of excitonic effects were observed in the low-energy regime of Landau-quantized graphene^3^,^4^. Furthermore, we assume an impurity-induced broadening of the LLs calculated in a self-consistent Born approximation^31^,^33^, where a reasonable strength of the impurity scattering is chosen^32^,^33^ yielding a broadening of approximately 4 meV. Details of the calculations can be found in the supplementary material, including the tight-binding description of graphene in a magnetic field, the matrix elements, and explicit expressions for the scattering rates 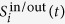.

**Figure 1:**
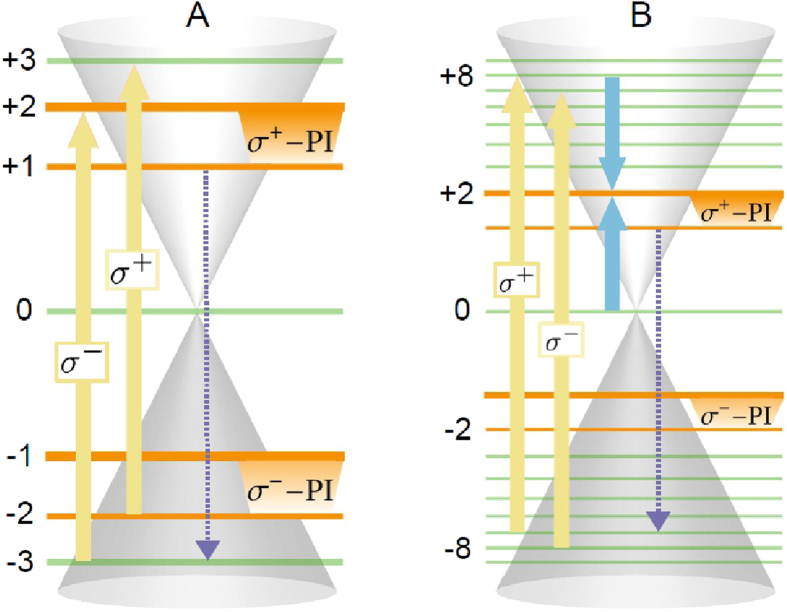
Schematic illustration of the population inversion. Graphene’s energetically lowest Landau levels (LLs) with the Dirac cone in the background illustrating different schemes to achieve population inversion between LL_+1_ and LL_+2_ (*σ*^+^-PI) and between LL_−2_ and LL_−1_ (*σ*^*−*^-PI), respectively. Scheme (**A**) is based solely on optical pumping (yellow arrows), which creates PI by populating LL+2 and depopulating LL−2. Scheme (**B**) exploits efficient Auger scattering (blue arrows) between the equidistant LLs + 8,+2 and 0 to redistribute optically pumped electrons (holes) from LL_+8_ (LL_−8_) to LL_+2_ (LL_−2_). To allow for continuous laser action, phonon-assisted relaxation channels are needed that connect all LLs involved in the laser system (purple-dotted arrows). For reasons of clearity, only carrier-carrier and carrier-phonon scattering channels corresponding to the *σ*^+^-PI in the conduction band are illustrated.

**Figure 2:**
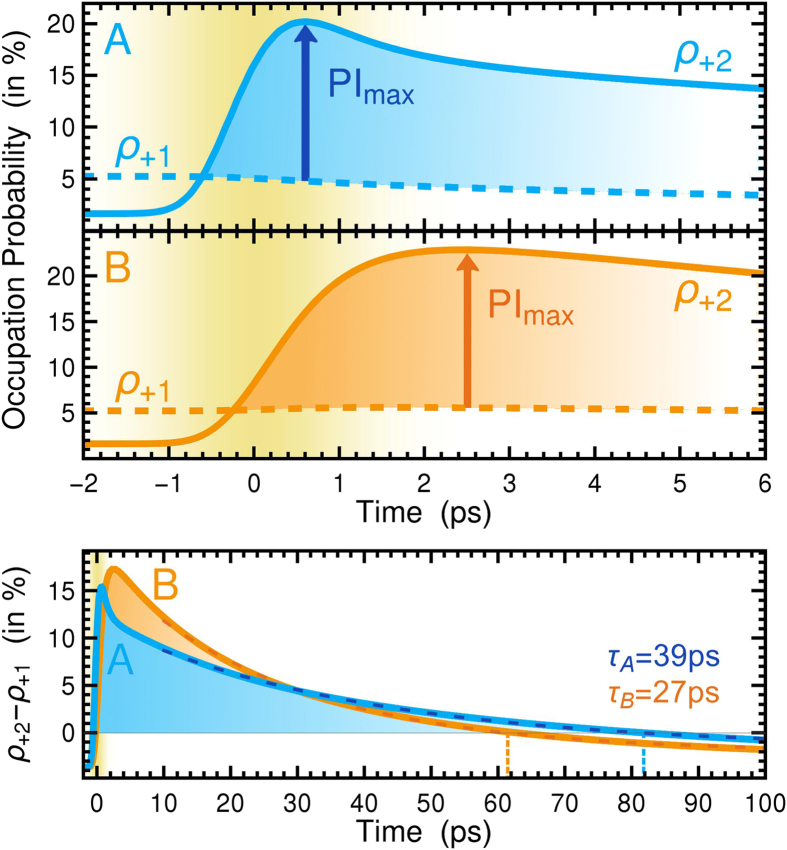
Dynamics of the carrier occupation. The temporal evolution of the occupations *ρ*_+1_ and *ρ*_+2_ in the upper panel for the PI schemes A and B (depicted in Fig. 1) illustrate the occurrence of a pronounced population inversion defined by PI = *ρ*_+2_ − *ρ*_+1_ > 0 (blue and orange shaded areas) **at a magnetic field of** 4T. The lower panel shows the temporal evolution of the PI featuring an ultrafast build-up and a slow decay on a ps time-scale. The vertical dashed lines mark the points in time where the PI vanishes (PI = 0) in the respective scheme. The yellow areas in the background illustrate the width of the optical excitation.

We investigate the carrier dynamics in graphene in the presence of an external magnetic field of *B* = 4T. We consider the system to be at room temperature with initial Fermi-Dirac distributed occupations that are optically excited by a pump pulse with a width of 1 ps, a pump fluence of *ε*_pf_ = 1*μ*Jcm^−2^, and an energy matching the pumped LL transitions of the respective PI scheme, cf. Fig. 1. In order to keep the excitation energy-dependent pulse area constant, the pump fluence in scheme B is increased to *ε*_pf_ = 2.27*μ*Jcm^−2^ (for details see Ref. 34). Solving the graphene Bloch equations (Eqs (1) and (2)) yields the temporal evolution of *p*_*ij*_ and *ρ*_*i*_ allowing us to investigate the interplay between optical transitions on the one side and carrier-carrier and carrier-phonon scattering processes on the other side. Since neutral Landau-quantized graphene is symmetric for electrons and holes, we focus the discussion on the *σ*^+^-PI in the conduction band. Figure 2 illustrates the time-dependent occupations of LL_+1_ and LL_+2_ (upper panel) as well as the resulting population inversion (lower panel). In both PI schemes, *ρ*_+1_(*t*) changes only slightly, while *ρ*_+2_(*t*) shows a fast increase on a sub-picosecond time scale during and shortly after the optical excitation (illustrated by the yellow area in the background) followed by a slow decay on a picosecond time scale. We find a long-lived population inversion that is defined by


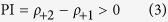


and represented by the respective areas between *ρ*_+1_ and *ρ*_+2_ in Fig. 2. Exponential fits to the temporal evolutions of PI for schemes A and B (dashed lines in lower panel of Fig. 2) reveal their decay times *τ*_*A*_ = 39 ps and *τ*_*B*_ = 27 ps. Since scheme A provides a straightforward approach to induce PI as a direct consequence of the optical excitation, the maximal value 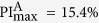 is reached already during pumping. In scheme B, on the other hand, Coulomb-scattering is needed to induce PI by redistributing the optically excited charge carriers, consequently, the build-up time is longer and its maximum 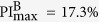 is reached with a delay of a few picoseconds. While both PI schemes are suited to create a significant PI with a rather long decay time, the advantage of scheme B is its additional fourth level making it a potentially better laser system which is reflected by its higher maximal PI value (cf. Fig. 1). As a consequence, optically excited charge carriers in LL_+8_ scatter down to LL_+2_ already during pumping which reduces the pumping saturation and allows the excitation of more charge carriers. The cost of the enhanced maximal PI is a faster decay resulting from the greater complexity of PI scheme B involving more LLs and therefore opening up more possible phonon-assisted decay channels: In scheme A the PI vanishes after ~82 ps, while in scheme B the PI lasts for ~62 ps.

**Figure 3:**
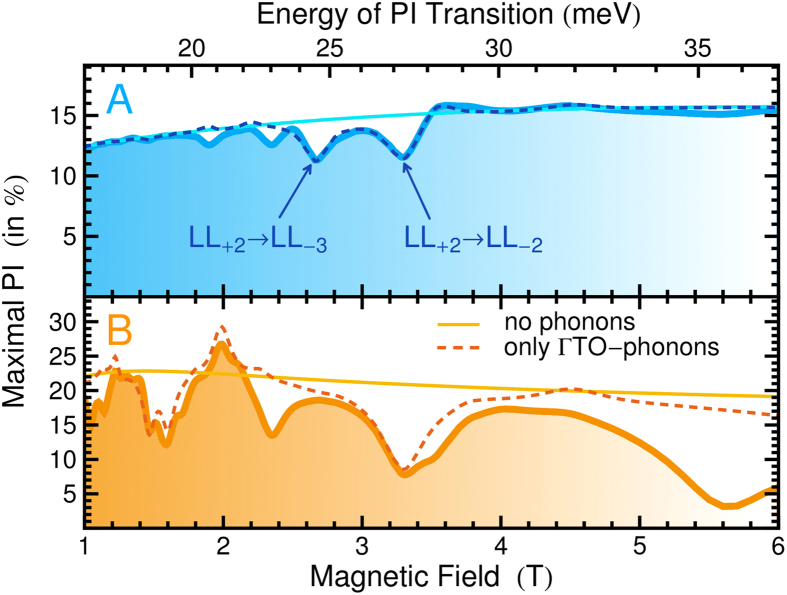
Magnetic field dependence. Maximal population inversion in dependence of the magnetic field (tick lines) for the PI schemes (**A**) and (**B**). The thin lines represent the dynamics when the electron-phonon scattering is switched off, and the dashed lines are obtained considering only ΓTO-phonons.

To obtain more insights into the underlying elementary processes, we investigate the maximal PI as a function of the magnetic field, cf. Fig. 3. For this investigation, we determine the maximal PI at different magnetic fields *B*, while the pulse area is held constant by scaling the pump fluence linearly with *B* (for details see Ref. 34). The energy of the PI transition *ε*_+2_ − *ε*_+1_ is tunable via the magnetic field and is given on the upper axis. The dynamics without phonons (thin lines) shows a weak dependence on the magnetic field: At higher *B*, the LLs are shifted to higher energies, thereby reducing the initial occupations in the conduction band, in particular *ρ*_+1_ decreases resulting in a stronger PI. However, at the same time the scattering generally becomes more efficient with increasing magnetic fields, since the degeneracy^34^ of LLs scales with *B*. This enhances the dephasing *γ*(*t*) of *p*_*ij*_ (cf. Eq. (2)) and reduces the pumping efficiency and consequently also the maximal PI. These counteracting effects nearly balance each other out resulting in a very weak dependence on the magnetic field. Switching on phonon-induced scattering, the dependence qualitatively changes and pronounced peaks and dips emerge in the *B*-dependence of the maximal PI (thick lines). They indicate magnetic fields that fulfill the resonance condition between the energy of an optical phonon and inter-Landau level transitions involved in the respective PI scheme. This is further evidenced by showing the dynamics that only includes ΓTO-phonons (dashed lines), where the number of peaks and dips is clearly reduced. In scheme A, two main resonances are present at the magnetic fields *B* = 2.67T and *B* = 3.30T. The first corresponds to the transition LL_+2_ → LL_−3_, while the second coincides with the transition LL_+2_ → LL_−2_, both reducing the PI by providing direct decay channels of the PI. The *B*-dependence in scheme B is more complex, since more LLs are involved (cf. Fig. 1) and hence more resonances occur. The three distinct dips at *B* = 1.45T, *B* = 1.60T, and *B* = 3.30T correspond to the transitions LL_+2_ → LL_−8_, LL_+2_ → LL_−7_, and LL_+2_ → LL_−2_ (and at the same time LL_+8_ → LL_0_), respectively. For reasons of clarity, symmetric hole transitions are omitted for the discussion. Interestingly, phonon-induced scattering can also increase the PI, as can be seen at *B* = 1.22T and *B* = 1.99T. At these magnetic field strengths, the energy of the ΓTO-phonon is in resonance with the transitions LL_+4_ → LL_−7_ and LL_+1_ → LL_−7_, respectively. The former case positively affects the pumping, while in the latter case the PI is not only increased through the depletion of LL_+1_. It also couples the lower laser level LL_+1_ with the ground state LL_−7_ from which electrons are excited and thus allows an electron to perform cycles in the four-level system opening up the possibility of continuous laser action **(cf.** Fig. 1): First it is excited from LL_−7_ to LL_+8_, from where it scatters down and accumulates in LL_+2_, before it participates in a stimulated emission event that transfers it to LL_+1_. Now, a phonon with the appropriate energy can bring the electron back to LL_−7_. The resonance condition is fulfilled for the ΓTO, ΓLO, and KTO-phonon modes at the magnetic fields *B* = 1.99T, *B* = 2.11T, and *B* = 1.41T, respectively. A magnetic field of *B* = 2T seems to be optimal, since no interfering resonances with other inter-LL transitions occur (cf. Fig. 3).

**Figure 4:**
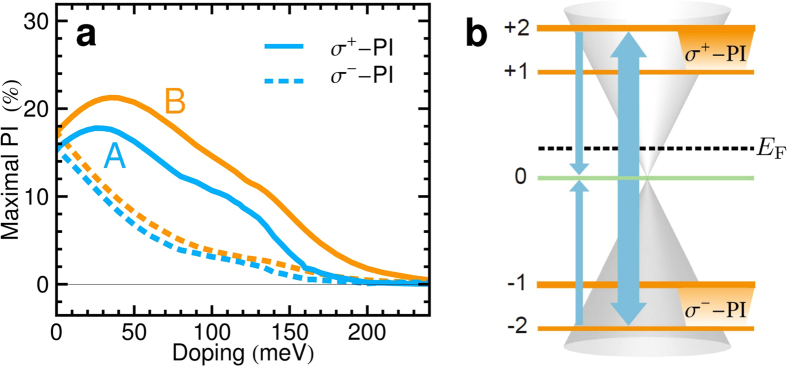
Doping dependence. Maximal PI between LL_+1_ and LL_+2_, and between LL_−2_ and LL_−1_ as a function of doping for *B* = 4T (**a**). The sketch (**b**) illustrates that Coulomb-induced scattering (thick arrows) prevails against its inverse process (thin arrows) resulting in an asymmetry of the two PI transitions.

Next, we explore the doping dependence of the PI, which opens up the possibility to control it by the application of a gate voltage resulting in a shift of the Fermi energy away from *E*_F_ = 0. This breaks the electron-hole symmetry and allows to tune the relative population inversion between the two inversely polarized LL transitions LL_±2_ → LL_±1_, cf. Fig. 4a. While a small positive Fermi energy gives rise to an increase of the *σ*^+^- PI, the impact on the *σ*^−^- PI is opposite. This behavior can be attributed to new scattering channels that are forbidden under electron-hole symmetry, but arise as soon as this symmetry is broken. For simplicity, we focus on the simple PI scheme A in the following: An up-shift of the Fermi energy results in a less efficient pumping of the transition LL_−3_ → LL_+2_ due to an enhanced Pauli blocking in comparison to the transition LL_−2_ → LL_+3_, since LL_+2_ becomes thermally occupied. According to this, *σ*^+^- PI should be suppressed, while *σ*^−^- PI is expected to be enhanced. Interestingly, we observe the opposite behavior, as shown in Fig. 4a. To understand this, we consider the energy-conserving Coulomb process involving the transitions LL_0_ → LL_+2_ and LL_0_ → LL_−2_ (outward scattering), which cancels out in an electron-hole symmetric system that also exhibits the inverse process (inward scattering: LL_+2_ → LL_0_ and LL_−2_ → LL_0_) occurring with the same probability. However, due to the asymmetric pumping and a more than half-filled LL_0_ in a n-doped sample, the Coulomb-induced outward scattering prevails over the inward scattering, cf. Fig. 4b. As a result, *σ*^+^- PI is enhanced, while *σ*^−^- PI is suppressed, as observed in Fig. 4a. Shifting the Fermi energy further away from the neutral position, the PI of both transitions decreases, which can be readily understood considering the initial occupations. When the Fermi energy reaches the vicinity of LL_+1_, its initial occupation *ρ*_+1_(*t*_0_) is considerably increased counteracting the build-up of a population inversion. The PI in both schemes shows a similar dependence of the doping.

Finally, we propose a pump-probe experiment to test our predictions at sufficiently low temperatures, so that the initial occupations *ρ*_+1_(*t*_0_) and *ρ*_+2_(*t*_0_) are nearly zero: Exciting Landau-quantized graphene according to one of the PI schemes A or B, cf. Fig. 1, with a probe pulse measuring the *σ*^+^- PI absorption, a positive differential transmission signal (DTS) indicates a faster increase of *ρ*_+2_ in comparison to *ρ*_+1_ and consequently would provide strong evidence for the occurrence of gain. More generally, in the presence of gain, the real part of the optical conductivity (which is proportional to the absorption) should become negative at the energy matching the transition LL_+1_ → LL_+2_. Based on our calculations, the optimal experimental conditions to measure gain in Landau-quantized graphene are expected at a magnetic field of *B* = 2T and a linearly polarized optical excitation with an energy of about *ε*_+8_ − *ε*_−7_ = 281 meV (depending on the exact value of the Fermi velocity, cf. Eq. 3 of the Supplementary material) corresponding to excitation scheme B (cf. Fig. 1).

In conclusion, based on microscopic calculations we predict the occurrence of a pronounced population inversion in Landau-quantized graphene. We demonstrate that controlling the magnetic field and the doping allows to tune the energy as well as the polarization of the emitted radiation. We show that carrier-phonon scattering can be exploited to boost the effect and to even open the way to continuous wave laser operation. Our microscopic insights into the carrier dynamics in Landau-quantized graphene will guide future experiments towards the design of graphene-based Landau level lasers and THz emitters.

## Methods

The microscopic modeling of the carrier dynamics has been performed within the formalism of density matrix theory^29^,^35^,^36^. The magnetic field has been implemented into the equations by applying the Peierls substitution^11^,^31^. A detailed description of the calculations including the electronic dispersion in the presence of a magnetic field as well as the coupling elements determining the carrier-light, carrier-carrier, and carrier-phonon interaction can be found in the supplementary material.

## Additional Information

**How to cite this article**: Wendler, F. and Malic, E. Towards a tunable graphene-based Landau level laser in the terahertz regime. *Sci. Rep.* **5**, 12646; doi: 10.1038/srep12646 (2015).

## Supplementary Material

Supplementary Information
