# Supplementary material for: Towards a tunable graphene-based Landau level laser in the terahertz regime
Source: Sci Rep. 2015 Jul 29;5:12646. doi: 10.1038/srep12646 (PMC4518241; doi:10.1038/srep12646)
Supplement: Supplementary Information [file srep12646-s1.pdf]

# Towards a tunable graphene-based Landau level laser in the terahertz regime – Supplementary material

Florian Wendler and Ermin Malic*

*Department of Applied Physics, Chalmers University of Technology, SE-412 96 Göteborg, Sweden*

To evaluate the Bloch equations for Landau-quantized graphene, cf. Eqs. (1)-(2) in the main part, we need to calculate the electronic dispersion, the wave function, and the matrix elements determining the many-particle interaction. This is discussed in detail below.

## 1. Wave function and dispersion in Landau-quantized graphene

The magnetic field $\mathbf{B} = \nabla \times \mathbf{A}$, which shall point into the z-direction $\mathbf{B} = (0, 0, B)$, is introduced by applying the Peierls substitution

$$\mathbf{p} \rightarrow \boldsymbol{\pi} = \mathbf{p} + e_0 \mathbf{A}(\mathbf{r}) \tag{1}$$

to the effective Hamiltonian of graphene in the low-energy regime

$$H^{\xi}_{\mathbf{q}} = \xi \hbar v_{\mathrm{F}} \begin{pmatrix} 0 & q_x - i q_y \\ q_x + i q_y & 0 \end{pmatrix}, \tag{2}$$

with the canonical momentum $\mathbf{p} = \hbar \mathbf{q}$, the kinetic momentum $\boldsymbol{\pi}$, the vector potential $\mathbf{A}(\mathbf{r})$, the Fermi velocity $v_{\mathrm{F}} = 1\,\mathrm{nm/fs}$ [1], and the valley index $\xi = \pm 1$ [2]. The Schrödinger equation is readily solved and yields the Landau level spectrum

$$\epsilon_{\lambda n} = \lambda v_{\mathrm{F}} \sqrt{2 \hbar e_0 B n}, \tag{3}$$

and the spinor

$$|\boldsymbol{\psi}\rangle = \alpha_n \begin{pmatrix} -\Theta(n-1)\, i \xi \lambda |n-1, m\rangle \\ |n, m\rangle \end{pmatrix}, \; \alpha_n = \begin{cases} 1 & , n = 0 \\ 1/\sqrt{2} & , n > 0 \end{cases}, \tag{4}$$

where $e_0$ is the elementary charge, $\lambda = \pm 1$ is the band index, $n = 0, 1, 2, \ldots$ **is the Landau level index**, and $m$ is a quantum number that can be associated with the position of

the cyclotron orbits in the graphene plane [2]. The tight-binding wave function is a linear combination of wave functions of the two sublattices A and B and reads

$$\Psi(\mathbf{r}, \lambda n, m, \xi) = \frac{1}{\sqrt{N}} \sum_{l \in \{\mathrm{A,B}\}} \sum_{\mathbf{R}_l} c_l(\mathbf{R}_l, \lambda n, m, \xi) e^{i\xi \mathbf{K}\cdot\mathbf{R}_l} \phi(\mathbf{r} - \mathbf{R}_l), \tag{5}$$

with the coefficients $c_\mathrm{A}$ and $c_\mathrm{B}$ that are given by the spatial representation of the spinor in Eq. 4 through the relation

$$\langle \mathbf{R} | \boldsymbol{\psi} \rangle = \begin{pmatrix} c_\mathbf{A}(\mathbf{R}, \lambda n, m, \xi) \\ c_\mathbf{B}(\mathbf{R}, \lambda n, m, \xi) \end{pmatrix}, \tag{6}$$

and where $\phi(\mathbf{r} - \mathbf{R}_l)$ denotes the $\mathrm{p_z}$–orbital of the carbon atom at position $\mathbf{R}_l$. The explicit form of $\langle \mathbf{R} | n, m \rangle$ is given by [3]

$$\langle \mathbf{R} | n, m \rangle = \frac{1}{l_B} \sqrt{\frac{A}{2\pi}} i^{|n-m|} \sqrt{\frac{\min(n,m)!}{\max(n,m)!}} e^{-R^2/(4l_B^2)} \left( \frac{R}{\sqrt{2} l_B} \right)^{|n-m|} e^{i(n-m)\varphi} L_{\min(n,m)}^{|n-m|} \left( \frac{R^2}{2l_B^2} \right), \tag{7}$$

with the magnetic length $l_B = \sqrt{\hbar/(e_0 B)}$.

## 2. Matrix elements

### 2.1 Optical matrix element

An analytic expression of the optical matrix element $\mathbf{M}_{i,f} = \int \mathrm{d}\mathbf{r} \Psi_f^*(\mathbf{r}) \nabla \Psi_i(\mathbf{r})$ is obtained using the wave function from Eq. 5 and reads [4]

$$\mathbf{M}_{i,f} = \alpha_{n_i} \alpha_{n_f} \frac{3M}{2} \left[ -\lambda_f \left( \hat{\mathbf{e}}_x + i\xi_f \hat{\mathbf{e}}_y \right) \delta_{n_f, n_i+1} + \lambda_i \left( \hat{\mathbf{e}}_x - i\xi_i \hat{\mathbf{e}}_y \right) \delta_{n_f, n_i-1} \right] \delta_{\xi_f, \xi_i} \delta_{m_f, m_i}, \tag{8}$$

with the compound index $i = (n_i, m_i, \lambda_i, \xi_i)$, the unit vector in $j$–direction $\hat{\mathbf{e}}_j$, and the overlap $M = \langle \phi(\mathbf{r} + a_0/\sqrt{3}\hat{\mathbf{e}}_x) | \partial_x | \phi(\mathbf{r}) \rangle$, where $a_0 = 0.2461\,\mathrm{nm}$ is the lattice spacing [5]. The overlap $M$ can be expressed in terms of the Fermi velocity via the relation $M = m_0 v_\mathrm{F}/(3\hbar)$ [4] which removes the dependence of the free electron mass $m_0$ from the Rabi frequency $\Omega_{ij} = \frac{e_0}{m_0} \mathbf{M}_{ij} \cdot \mathbf{A}(t)$. The Kronecker deltas express the graphene-specific optical selection rules

$n \to n \pm 1$ allowing transitions only between Landau levels **with an index $n$ differing by 1. These inter-Landau level transitions can be induced using circularly polarized radiation, where $\sigma^{\pm}$-polarized light corresponds to $\Delta n = \pm 1$ respectively [6, 7]. When a combination of both circular polarization directions is used, e.g. in a linearly polarized excitation field, both transitions ($\Delta n = \pm 1$) are pumped at the same time.**

### 2.2 Coulomb matrix element

The double integration occurring in the Coulomb matrix element

$$V_{34}^{12} = \int \mathrm{d}\mathbf{r} \int \mathrm{d}\mathbf{r}' \Psi_3^*(\mathbf{r}) \Psi_4^*(\mathbf{r}') V_{\text{Coul}}(\mathbf{r} - \mathbf{r}') \Psi_2(\mathbf{r}') \Psi_1(\mathbf{r}) \tag{9}$$

is simplified introducing the Fourier transformation

$$V_{34}^{12} = \sum_{\mathbf{q}} V_{\mathbf{q}} \Gamma_{13}(\mathbf{q}) \Gamma_{24}(-\mathbf{q}), \tag{10}$$

which results in the splitting into two separate integrals $\Gamma_{13}(\mathbf{q})$ and $\Gamma_{24}(-\mathbf{q})$. Here, $V_{\mathbf{q}} = e_0^2/(2\epsilon_0 \epsilon_r A q)$ is the Fourier transform of the Coulomb interaction with the vacuum and relative permittivities $\epsilon_0$ and $\epsilon_r$, and the area of graphene $A$. We consider graphene on a SiC substrate with a relative permittivity of $\epsilon_r \approx (\epsilon_{\text{SiC}} + \epsilon_{\text{air}})/2 \approx 3.3$ [8]. The integrals $\Gamma_{if}(\mathbf{q}) = \int \mathrm{d}\mathbf{r}\, \Psi_f^*(\mathbf{r}) e^{i\mathbf{q}\mathbf{r}} \Psi_i(\mathbf{r})$ are evaluated using the tight-binding wave functions (Eq. 5) yielding

$$\begin{aligned} V_{34}^{12} = {} & \delta_{\xi_1,\xi_3} \delta_{\xi_2,\xi_4} \alpha_{n_1} \alpha_{n_2} \alpha_{n_3} \alpha_{n_4} \frac{A}{4\pi^2} \int_0^{\infty} \mathrm{d}q \int_0^{2\pi} \mathrm{d}\varphi V_{\mathbf{q}} q \\ & \times \left[ \lambda_1 \lambda_3 \langle n_3 - 1, m_3 | e^{i\mathbf{q}\mathbf{r}} | n_1 - 1, m_1 \rangle + \langle n_3, m_3 | e^{i\mathbf{q}\mathbf{r}} | n_1, m_1 \rangle \right] \\ & \times \left[ \lambda_2 \lambda_4 \langle n_4 - 1, m_4 | e^{-i\mathbf{q}\mathbf{r}} | n_2 - 1, m_2 \rangle + \langle n_4, m_4 | e^{-i\mathbf{q}\mathbf{r}} | n_2, m_2 \rangle \right], \end{aligned} \tag{11}$$

with the momentum transfer in polar coordinates $\mathbf{q} = (q, \varphi)$, and the form factors [2]

$$\begin{aligned} \langle nm | e^{\pm i\mathbf{q}\mathbf{r}} | n'm' \rangle = {} & e^{-\frac{\hbar q^2}{2e_0 B}} \sqrt{\frac{\min(m', m)!}{\max(m', m)!}} \sqrt{\frac{\min(n', n)!}{\max(n', n)!}} \left( \pm i q \sqrt{\frac{\hbar}{2e_0 B}} \right)^{|m-m'|+|n-n'|} \\ & \times \left( e^{i\varphi} \right)^{m-m'-n+n'} L_{\min(m',m)}^{|m-m'|} \left( \frac{\hbar q^2}{2e_0 B} \right) L_{\min(n',n)}^{|n-n'|} \left( \frac{\hbar q^2}{2e_0 B} \right). \end{aligned} \tag{12}$$

Note that the angular integration in Eq. 11 yields a Kronecker delta $\delta_{n_1-m_1+n_2-m_2,n_3-m_3+n_4-m_4}$ expressing the angular momentum conservation of Coulomb scattering. Dynamical screening is taken into account by replacing

$$V_{\mathbf{q}} \rightarrow \frac{V_{\mathbf{q}}}{\epsilon(\mathbf{q},\omega)} \tag{13}$$

in Eq. 11, where the energy in the dielectric function $\epsilon(\mathbf{q},\omega) == 1 - V_{\mathbf{q}}\Pi^0(\mathbf{q},\omega)$ is defined as $\hbar\omega = \epsilon_1 - \epsilon_3$ [9], and $\Pi^0(\mathbf{q},\omega)$ denotes the polarizability which is given by

$$\Pi^0(\mathbf{q},\omega) = 4\sum_{\lambda\lambda'}\sum_{nn'} \frac{n_{\mathrm{FD}}(\epsilon_{\lambda n}) - n_{\mathrm{FD}}(\epsilon_{\lambda' n'})}{\epsilon_{\lambda n} - \epsilon_{\lambda' n'} + \hbar\omega + i\Gamma} \left|F_{\lambda n,\lambda' n'}(\mathbf{q})\right|^2 \tag{14}$$

in the random phase approximation [2, 10]. Here, $n_{\mathrm{FD}}$ is the Fermi-Dirac distribution, and a constant broadening $\Gamma = 4\,\mathrm{meV}$ is introduced. The latter is a Landau level broadening induced by electron-impurity scattering. The form factor $F_{\lambda n,\lambda' n'}(\mathbf{q})$ reads [2]

$$F_{\lambda n,\lambda' n'}(\mathbf{q}) = \sqrt{\frac{(1-\delta_{n,0})\,(1-\delta_{n',0})}{4}}\, f_{n-1,n'-1}(\mathbf{q}) + \lambda\lambda' \sqrt{\frac{(1+\delta_{n,0})\,(1+\delta_{n',0})}{4}}\, f_{n,n'}(\mathbf{q}), \tag{15}$$

with

$$f_{n,n'}(\mathbf{q}) = e^{-\frac{\hbar q^2}{4e_0 B}} \sqrt{\frac{\min(n,n')!}{\max(n,n')!}} \left(-iq\frac{l_B}{\sqrt{2}}\right)^{\max(n,n')-\min(n,n')} L_{\min(n',n)}^{\max(n,n')-\min(n,n')}\left(\frac{l_B^2 q^2}{2}\right). \tag{16}$$

### 2.3 Phonon matrix elements

The carrier-phonon matrix element $g^{i,f}_{\mathbf{p},\mu} = \int d\mathbf{r}\Psi^*_f(\mathbf{r})V_{\text{c-ph}}(\mathbf{p})\Psi_i(\mathbf{r})$ depends on the potential $V_{\text{c-ph}}(\mathbf{p})$ that was explicitly calculated by Ando in the long-wavelength limit [11]. Plugging the wave function Eq. 5 into the above expression with the potential $V_{\text{c-ph}}(\mathbf{p})$ of the respective optical phonon mode yields

$$g^{i,f}_{\mathbf{p},\Gamma\text{-O}} = 3\sqrt{2}i\xi\beta\alpha_{n_i}\alpha_{n_f}\frac{\hbar^2 v_F}{a_0^2\sqrt{MA\epsilon_{\mathbf{p},\Gamma-\mathrm{O}}}}\Big[\lambda_i e^{i\xi\varphi_{\mathbf{p}}}\langle n_f, m_f|e^{i\mathbf{pr}}|n_i-1, m_i\rangle \\ \pm\lambda_f e^{-i\xi\varphi_{\mathbf{p}}}\langle n_f-1, m_f|e^{i\mathbf{pr}}|n_i, m_i\rangle\Big], \tag{17}$$

$$\left|g^{i,f}_{\mathbf{p},\text{K-O}}\right|^2 = 2\left\langle g^2_{\text{K-O}}\right\rangle_{\mathrm{DFT}} c^2_{n_i}c^2_{n_f}\Big|e^{-i\varphi_{\mathbf{p}}}\langle n_f, m_f|e^{i\mathbf{pr}}|n_i, m_i\rangle \\ \pm\lambda_i\lambda_f e^{-i\varphi_{\mathbf{p}}}\langle n_f-1, m_f|e^{i\mathbf{pr}}|n_i-1, m_i\rangle\Big|^2, \tag{18}$$

with the coupling parameter $\beta \approx 2$ [11], the phonon momentum transfer in polar coordinates $\mathbf{p} = (p, \varphi_{\mathbf{p}})$, the graphene mass density $M = 7.6 \times 10^{-8}\,\mathrm{gcm}^{-2}$, and the lattice spacing $a_0 = 0.2461\,\mathrm{nm}$ [5]. Furthermore, the + sign refers to the longitudinal modes (ΓLO, KLO) and the - sign to the transverse modes (ΓTO, KTO). While analytic expressions are obtained in the case of Γ phonons, the carrier-phonon interaction strengths $\langle g^2_{\mathrm{KTO}}\rangle_{\mathrm{DFT}} = 0.0994\,\mathrm{eV}^2 \cdot A_{\mathrm{uc}}/A$, and $\langle g^2_{\mathrm{KLO}}\rangle_{\mathrm{DFT}} = 0.00156\,\mathrm{eV}^2 \cdot A_{\mathrm{uc}}/A$ are based on numerical calculations within density functional theory (DFT) that where performed by Piscanec et. al [12]. Here, $A_{\mathrm{uc}} = \sqrt{3}a_0^2/2$ is the unit cell area and $A$ is the area of graphene which cancels after performing the momentum sum in the scattering rates. Note that the interaction strengths in the case of the Γ phonons are also in agreement with the corresponding DFT calculations [12]. The energies of the phonon modes are considered to be constant, i.e. $\epsilon_{\mathbf{p},\Gamma\mathrm{TO}} = 192\,\mathrm{meV}$, $\epsilon_{\mathbf{p},\Gamma\mathrm{LO}} = 198\,\mathrm{meV}$, $\epsilon_{\mathbf{p},\mathrm{KTO}} = 162\,\mathrm{meV}$, and $\epsilon_{\mathbf{p},\mathrm{KLO}} = 151\,\mathrm{meV}$ [13, 14]. Finally, the form factors $\langle n_f, m_f|e^{i\mathbf{pr}}|n_i, m_i\rangle$ are given by Eq. 12. The KLO–phonon mode is omitted in the numerical calculations, because its interaction strength is negligible compared to the three other modes. Since a phonon can absorb (or provide) any angular momentum, there are no selection rules for the carrier-phonon interaction and restrictions of the corresponding scattering channels are only imposed by the energy conservation.

## 3. Scattering rates

Many-particle scattering rates appearing in the Bloch equations, cf. Eqs. 1–2 in the main part of the paper, read in second-order Born-Markov approximation for the Coulomb interaction

$$S_f^{\mathrm{in}}(t)\Big|_{\mathrm{c,c}} = \frac{2\pi}{\hbar}\sum_{abc} V^{fa}_{bc}\left(V^{bc}_{fa} - V^{cb}_{fa}\right)(1-\rho_a)\,\rho_b\rho_c L_\Gamma\left(\triangle E_{fabc}\right) \tag{19}$$

$$S_i^{\mathrm{out}}(t)\Big|_{\mathrm{c,c}} = \frac{2\pi}{\hbar}\sum_{abc} V^{ia}_{bc}\left(V^{bc}_{ia} - V^{cb}_{ia}\right)\rho_a\,(1-\rho_b)\,(1-\rho_c)\,L_\Gamma\left(\triangle E_{iabc}\right), \tag{20}$$

and for the carrier-phonon interaction

$$S_f^{\mathrm{in}}(t)\Big|_{\mathrm{c,p}} = \frac{2\pi}{\hbar}\sum_{i}\sum_{\mathbf{p},\mu}\left|g^{i,f}_{\mathbf{p},\mu}\right|^2\rho_i\left[\left(n_{\mathbf{p},\mu}+1\right)L_\Gamma\left(\triangle E^{\mathrm{em}}_{if\mu}\right) + n_{\mathbf{p},\mu}L_\Gamma\left(\triangle E^{\mathrm{ab}}_{if\mu}\right)\right] \tag{21}$$

$$S_i^{\mathrm{out}}(t)\Big|_{\mathrm{c,p}} = \frac{2\pi}{\hbar}\sum_{f}\sum_{\mathbf{p},\mu}\left|g^{i,f}_{\mathbf{p},\mu}\right|^2(1-\rho_f)\left[\left(n_{\mathbf{p},\mu}+1\right)L_\Gamma\left(\triangle E^{\mathrm{em}}_{if\mu}\right) + n_{\mathbf{p},\mu}L_\Gamma\left(\triangle E^{\mathrm{ab}}_{if\mu}\right)\right], \tag{22}$$

with the Coulomb matrix element $V_{34}^{12}$, the carrier-phonon matrix element $g_{\mathbf{p},\mu}^{i,f}$, the phonon occupation $n_{\mathbf{p},\mu}$, the energy differences $\triangle E_{iabc} = \epsilon_i + \epsilon_a - \epsilon_b - \epsilon_c$ and $\triangle E^{\mathrm{em/ab}} = \epsilon_f - \epsilon_i \pm \epsilon_{\mathbf{p},\mu}$ for the emission (+) and absorption (−) of a phonon of the mode $\mu$. We assume a finite Landau level broadening induced by electron-impurity scattering that is expressed by a Lorentzian

$$L_\Gamma\left(\triangle E\right) = \frac{\Gamma}{\pi\left(\triangle E^2 + \Gamma^2\right)}. \tag{23}$$


[1] A. H. Castro Neto, F. Guinea, N. M. R. Peres, K. S. Novoselov, and A. K. Geim, Rev. Mod. Phys. **81**, 109 (2009).

[2] M. O. Goerbig, Rev. Mod. Phys. **83**, 1193 (2011).

[3] Y. Lozovik and A. Sokolik, Nanoscale Res. Lett. **7**, 134 (2012).

[4] K. M. Rao and J. E. Sipe, Phys. Rev. B **86**, 115427 (2012).

[5] S. Reich, J. Maultzsch, C. Thomsen, and P. Ordejón, Phys. Rev. B **66**, 035412 (2002).

[6] F. Wendler, A. Knorr, and E. Malic, "Ultrafast carrier dynamics in landau-quantized graphene," (accepted for publication in Nanophotonics).

[7] F. Wendler, H. Funk, M. Mittendorff, S. Winnerl, M. Helm, A. Knorr, and E. Malic, Proc. SPIE **9361**, 936105 (2015).

[8] E. Malic and A. Knorr, *Graphene and Carbon Nanotubes: Ultrafast Optics and Relaxation Dynamics* (Wiley-VCH, 2013).

[9] A. Tomadin, D. Brida, G. Cerullo, A. C. Ferrari, and M. Polini, Phys. Rev. B **88**, 035430 (2013).

[10] R. Roldan, M. O. Goerbig, and J.-N. Fuchs, Semicond. Sci. Technol. **25**, 034005 (2010).

[11] T. Ando, J. Phys. Soc. Jpn. **75**, 124701 (2006).

[12] S. Piscanec, M. Lazzeri, F. Mauri, A. C. Ferrari, and J. Robertson, Phys. Rev. Lett. **93**, 185503 (2004).

[13] E. Malic, T. Winzer, E. Bobkin, and A. Knorr, Phys. Rev. B **84**, 205406 (2011).

[14] J. Maultzsch, S. Reich, C. Thomsen, H. Requardt, and P. Ordejón, Phys. Rev. Lett. **92**, 075501 (2004).
